# Influence of Owners’ Attachment Style and Personality on Their Dogs’ (*Canis familiaris*) Separation-Related Disorder

**DOI:** 10.1371/journal.pone.0118375

**Published:** 2015-02-23

**Authors:** Veronika Konok, András Kosztolányi, Wohlfarth Rainer, Bettina Mutschler, Ulrike Halsband, Ádám Miklósi

**Affiliations:** 1 Department of Ethology, Eötvös Loránd University, Pázmány P. s. 1/c, H-1117 Budapest, Hungary; 2 MTA-DE “Lendület” Behavioural Ecology Research Group, Department of Evolutionary Zoology and Human Biology, University of Debrecen, Debrecen, Hungary; 3 Freiburg University of Education, Department of Public Health / Health Education, Freiburg, Germany; 4 Freiburg Institute for Animal-Assisted Therapy, Freiburg, Germany; 5 University of Freiburg, Department of Psychology, Neuropsychology, Freiburg, Germany; 6 MTA-ELTE Comparative Ethology Research Group, Budapest, Hungary; University of Portsmouth, UNITED KINGDOM

## Abstract

Previous research has suggested that owners’ attitude to their family dogs may contribute to a variety of behaviour problems in the dog, and authors assume that dogs with separation-related disorder (SRD) attach differently to the owner than typical dogs do. Our previous research suggested that these dogs may have an insecure attachment style. In the present study we have investigated whether owners’ attachment style, personality traits and the personality of the dog influence the occurrence of SRD in the dog. In an internet-based survey 1508 (1185 German and 323 Hungarian) dog-owners filled in five questionnaires: Demographic questions, Separation Behaviour Questionnaire (to determine SRD), Human and Dog Big Five Inventory and Adult Attachment Scale. We found that with owners’ higher score on attachment avoidance the occurrence of SRD in the dog increases. Dogs scoring higher on the neuroticism scale were more prone to develop SRD. Our results suggest that owners’ attachment avoidance may facilitate the development of SRD in dogs. We assume that avoidant owners are less responsive to the dog’s needs and do not provide a secure base for the dog when needed. As a result dogs form an insecure attachment and may develop SRD. However, there may be alternative explanations of our findings that we also discuss.

## Introduction

Dogs and humans have been living together for tens of thousands of years and during this time domestication might have predisposed dogs to form attachment relationships with humans [1]. Dog puppies are typically acquired by human families at age between 6 and 10 weeks and the human owner becomes readily the primary attachment figure for the puppy [2]. Dogs show functionally analogue behaviours to human infants in the Strange Situation Test [3–5], that is, they seek the proximity of the owner and show stress-response during separation from him/her. The proximity of the owner serves as a *secure base* for the dog for exploring the environment [4–7] and a *safe haven* in threatening situations [8] similarly as parents’ proximity is for infants.

Humans also have a disposition to form an attachment relationship with their dogs [9] which might be facilitated by dogs’ paedomorphic morphological and behavioural features [10]. Kubinyi at al. [11] found that 93.3% of the owners considered their dogs as family members. Actually, using the Repertory Grid [12] technique Berryman [13] found that pet owners (mostly dogs and cats) see their pets significantly more like „own child” than any other family member. In addition, owners use their dogs as a safe haven (to alleviate stress) [9] more than any other family members or friends, except for romantic partners [14].

### Separation stress and related disorders

Stress response to separation is adaptive both in animals and humans. According to Bowlby [15] the ultimate function of parent–offspring attachment is to protect against predators and maintain the supply of resources for offspring if they remain in proximity to the parent(s). However, typical maturation results in increased tolerance of separation. In human children, separation anxiety [16] become problematic and is diagnosed as a separation anxiety disorder if it exceeds (in intensity and in age) normative reactions to separations from caregivers and cause troubles in social functioning [17]. Separation anxiety disorder is marked by recurrent excessive distress when separated from the home or important others, permanent and excessive worry about losing the attachment figures, refusal of going to school or reluctance to going to sleep alone [17,18].

A functionally analogue behaviour problem exists in dogs, which is referred to as separation-related disorder (SRD) [19]. This phenomenon occurs in the owner’s absence or when the dog is prevented access to the owner. Owners of dogs with SRD complain most frequently about destructive behaviour displayed at home, excessive vocalization (often noticed by neighbours), or inappropriate elimination (urination/defecation, e.g. [20]). Further symptoms (which are less easily recognized) include autonomic signs such as hypersalivation or hyperventilation, increased and repetitive motor activity (e.g. pacing, circling), repetitive behaviour (e.g. over-grooming or self-mutilation), behavioural signs of depression such as withdrawal, inactivity or inappetence, gastrointestinal symptoms (vomiting, diarrhea) or escape behaviour that can result in self-trauma [20–22]. Thus, it seems that both in humans and dogs there are individuals who have lower threshold for the activation of the attachment system [15], and who show a separation response that is developmentally inadequate, has extreme degree, form and consequences.

### Attachment, caregiving behaviour and separation anxiety

Attachment and separation anxiety are related concepts. According to Bowlby [15], securely attached children have the confidence that the attachment figure will be available and accessible if needed, thus they are less anxious during separation. In order for the child to feel accessibility and availability the mother (parent) has to be sensitive and responsive to the child’s needs (e.g. responsive to the infants' cries, sensitive in initiating and terminating feeding, etc.). Sensitive and responsive parenting consists of synchrony, mutuality, emotional support, positive attitude and stimulation [23].

In parallel with the theoretical assumptions, researchers found that insecurely attached children are more prone to show separation anxiety than securely attached children [24,25]. Children’s attachment style and separation anxiety is associated with parents’ caregiving behaviour [26–28]. Specifically, maternal responsiveness/sensitivity seems to be the primary predictor of a child’s secure attachment [29–33] and separation anxiety [25].

Parents’ caregiving behaviour is influenced by their own attachment style („adult attachment”, [34]). Van Ijzendoorn [35] hypothesized that the parents’ representation of past and present attachment experiences (Bowlby’s “inner working model”, [15]) influences the degree of sensitivity and responsiveness with which the parent reacts to the child’s attachment signals. These hypotheses were supported by investigations showing that insecurely attached adults show less consistent responsiveness to their children’s needs (for a meta-analysis, see [35]). More precisely, avoidant/dismissing mothers are the ones who are less sensitive/ responsive than secure mothers, and this is especially true in stressful situations or with mothers experiencing more psychological distress [36,37]. It seems that preoccupied (anxious) mothers have deficiency not in sensitivity but rather in autonomy support/non-intrusiveness [36,38].

As attachment styles are associated with different personality traits [39,40], and personality affects parenting [41], mothers’ personality can also affect the child’s attachment style and behaviour problems [42]. In summary, higher extraversion, agreeableness, conscientiousness and lower neuroticism seem to be associated with more secure adult attachment ([39,40], or for a meta-analytic review see [43]) and a warmer and more sensitive parenting (e.g. [41,42], or a meta-analysis: [44]). Thus these traits are plausible candidates for influencing children’s attachment style and behaviour problems including separation anxiety. However, as far as we know, no study has been carried out on the association between parental personality and the child’s separation anxiety.

### The etiology of SRD in dogs: owners’ attitude, dogs’ attachment

As SRD is much less studied in family dogs, we have only few data on the etiology of the disorder. The potential causes mentioned in the literature include pathologic “over-attachment” or “hyper-attachment” to the owner (e.g. [20]), negative early experiences such as too early separation from the bitch, other traumatic experiences while left alone, change in family circumstances (for details see [45]) or heritable factors [46]. McCrave [47] reported an increased prevalence of SRD in mixed breed dogs. However, mixed breed dogs are represented in a large percentage among shelter dogs [48] and staying in a shelter can contribute to the development of SRD [47,49]. SRD is reported more often in male dogs than in females [50–52]. Mendl et al. [53] reported that dogs from the shelter with SRD show more „pessimistic” choice behaviour in a food search test.

In a previous study [50] we showed that dogs with SRD do not use the owner as a secure base. These dogs are very distressed upon separation and they cannot be easily calmed down by the return of the owner. During separation, they do not use an object substitution of the owner for self-reassurance, as typical dogs do. Thus, we assume that SRD dogs living in human families have an insecure attachment style, analogue to human C type (insecure, ambivalent/anxious) [54]. Note that this view is in contrast with the popular theory which holds that SRD dogs are “hyper-attached” to the owner. Two studies [50,55] reported that SRD dogs do not show more affection toward the owner (expressed by e.g. proximity to and body contact with the owner, eye-contact with the owner, fast tail-wagging) which contradicts the “hyper-attachment” theory.

Based on the functional analogy in attachment between dogs and children we assume that owners’ responsiveness and sensitivity to the dog’s needs influences the dog’s attachment style and separation-related disorder as in the case of human mother-child dyads. Experts in behavioural disorders agree that owners’ attitude to the dog may contribute to a variety of behaviour problems [56]. For example, time spent with the dog and shared activities with the owner correlates negatively with dogs’ behaviour problems (e.g. disobedience, aggression, nervousness, overexcitement, etc.) [57,58]. Owners’ anthropomorphic emotional involvement correlates with the dog’s aggression toward people [56]. Owners’ personality was also found to be associated with dogs’ behaviour problems: Owners of aggressive dogs were reported to be emotionally less stable, more disciplined and tense than owners of non-aggressive dogs [59]. Owners’ neuroticism was found to correlate with the degree of the dog’s displacement activities [56] and with dogs’ neuroticism [60]. However, only one study observed an indirect association of owners’ attitudes and the dog’s SRD: the prevalence of separation- related elimination is lower if the dog has been subjected to obedience training and if it does not sleep in the bedroom of the owner [61].

### Aims of the study

Our main aim was to reveal characteristics of owners that may increase the occurrence of SRD in their dogs, with special focus on owners’ attachment and personality. We were also interested in associations of dogs’ personality and SRD as it can contribute to the better understanding of this behaviour problem. This is an exploratory study because the data about the dogs’ personality and SRD stems from the owners and not from independent experimental observations. However, in a previous experimental study [50] we showed that owners perceive correctly the separation and greeting behaviour of their dog: dogs that were rated by their owner in a questionnaire to be more “anxious” during separation and “happier” at reunion, showed more activity and stress-related behaviour (e.g. whining, scratching of the door, etc.) during the separation phase of a behaviour test, and more affection toward the owner (e.g. contact with the owner, tail-wagging, etc.) during the greeting phase of the test. Additionally, dogs that were rated by their owners to have SRD showed more stress-related behavior during separation in the behaviour test than dogs without owner-reported SRD.

We assumed that people with insecure-avoidant attachment style (in the two-dimensional model of attachment [62] it is called “attachment avoidance”) were less responsive to their dogs’ needs. This can lead to insecure attachment, and – as a consequence—SRD in their dogs.

We predicted that with owner’s higher attachment avoidance the prevalence of SRD in the dog would increase; however, degree of owners’ attachment anxiety would have no effect on dogs’ SRD. We base this assumption on the human literature: avoidant mothers do have deficiency in sensitivity/responsiveness but anxious mothers do not (e.g. [36]).

We predicted that owners’ higher neuroticism will also contribute to higher occurrence of SRD (i) because higher neuroticism is associated with less warm and sensitive parenting and less secure adult attachment in case of human parents, and (ii) because higher neuroticism of the owner was found to be associated with their dogs’ behaviour problem and neuroticism. Finally, we predicted also that more neurotic dogs (given their increased proneness to stress reaction) would have more often SRD.

## Method

### Subjects

Two separate studies were performed in Hungary and Germany using the same methodology. 323 Hungarian and 1185 German owners (Hungarians: 96 men, 227 women; median age = 31, range: from 18 to 70; Germans: 105 men, 1080 women; median age = 42, range: from 18 to 76) of family dogs (various pure and mixed breeds; Hungarian dogs: 154 males, 169 females; median age = 3.2, range: from 1 to 14, n = 227, dog age is missing in 96 cases; German dogs: 542 males and 617 females, n = 1159, dog gender is missing in 26 cases; median age = 4.65, range: from 1 to 17, n = 1116, dog age is missing in 69 cases) filled out the questionnaires. The sample of the dogs was random and non-clinical. Dogs were not screened for the presence of SRD in advance.

Owners were recruited from the dog-owner database of the Department of Ethology (Hungary) / the Freiburg Institute of Animal-Assisted Therapy (Germany) via email, via Facebook (dog owner pages), internet forums and advertisement on our homepages. The criteria of inclusion were that the dog had to be at least 1 year old and had to live together with the owner for at least half a year. By filling out the questionnaires owners became entitled to participate in a lottery in which they could win two dog-toys (Hungary) / twenty shopping coupons (20 Euros) for a pet food shop (Germany).

Due to practical reasons (communication problem with the owners) not all questionnaires were filled out by all Hungarian owners: 323 owners filled out the Adult Attachment Scale, 201 owners filled out the Big Five Inventory, and 201 owners filled out the Dog Big Five Inventory. All three questionnaires were filled out by 200 Hungarian owners. All of the German owners filled out all questionnaires.

### Ethics statement

The conducted research was neither physically nor emotionally demanding. The filling out of the questionnaires was anonym so the study does not violate respondents' privacy. The research was undertaken with great care, e.g. by ensuring the privacy and confidentiality of subjects, explaining the research process to them and assuring them of using the data only for scientific purposes. In addition, we consulted with the institutional review board (Ethical Committee of Eötvös Loránd University). They provided a written ethical approval for the study and a written statement that there is no need for the approval of higher ethics committees.

Informed written consent was not obtained from the participants because the data were anonymized at collection (although elements of informed consent were included in the introductory letter of the questionnaire). The Ethical Committee was aware of the consent procedure. The data presented here are not publicly available. However, a copy of the fully-anonymized dataset is available from the first author upon request.

### Materials and procedure

The Hungarian data collection began in December 2011 and ended in February 2012. The German data collection lasted from November 2012 until January 2013. Subjects filled out the questionnaires on an online interface and it took approximately half an hour. They were allowed to fill in the questionnaires at any place with internet access. The questionnaires had to be filled in at once as subjects could not save them for subsequent editing.

The following five questionnaires were used:

*Demographic questions* (see S1 Appendix): We asked owners about their gender and age, and about the breed, sex and age of the dog, and about how long they have been living together.

*Separation Behaviour Questionnaire* (see S2 Appendix): We asked whether the owner thinks that his/her dog has a separation-related disorder (owner reported SRD: Yes/No question, SRD hereafter) and we asked questions about nine specific behavioural symptoms characteristic of SRD (based on [20,21]). These behavioural symptoms were whining, barking, howling, salivation, urination, defecation, destruction, trembling and agitation (in more details, see S2 Appendix). Owners had to rate how frequently (1-never, 2-rarely, 3-frequently, 4-always/almost always) they experience the given symptom in the dog in those situations when they leave the dog alone or when they just happen to leave. We also offered an “I do not know” choice for each question. However, such answers were rare (1% of all questions) so we considered these answers as missing values.

*Adult Attachment Scale* (AAS [63]; see S3 Appendix; the Hungarian translation can be found in [64]; the German translation in [65]): the questionnaire contains 18 items in the original English and in the Hungarian versions, and 15 items in the German version. The item reduction in the German version was done because of descriptive, psychometric and content/thematic considerations [65]. AAS is based on a dimensional view of attachment and it originally contains three subscales: *closeness* (the degree to which a person is comfortable with closeness and intimacy), *dependence* (the extent to which a person feels he/she can depend on others or expect them to be available when needed) and *anxiety* (the extent to which a person is worried about being abandoned or unloved).

From these three subscales Collins [62] derived two main scales: *anxiety* (six items in the original English and in the Hungarian versions and five items in the German version) and *avoidance* (12 items in the original English and in the Hungarian versions and 10 items in the German version). We used these two derived scales in the analysis. *Avoidance* means attachment avoidance, and it is the reverse of the original *dependence* and *closeness* scales. The advantage of using these two dimensions is that they fit to other attachment models (e.g. [66,67]). Items are rated by the subjects on a 5-point Likert scale from 1 (not at all characteristic) to 5 (very characteristic).

*Big Five Inventory* (BFI, [68]; see S4 Appendix; Hungarian translation: [69]; German translation: [70,71]): the 44-item questionnaire is based on a framework of human personality, namely the Five Factor Model or the Big Five Theory [72–75] which holds that the personality can be described along five adjective factors or dimensions. For each item, the owners had to score themselves using a 5-point scale (from disagree strongly to agree strongly). The questionnaire contains five factors: *extraversion* (e.g. assertive, unreserved, sociable), *neuroticism* (e.g. anxious, nervous, depressed), *agreeableness* (e.g. kind, warm, trusting, cooperative), *conscientiousness* (e.g. persistent, self-disciplined, diligent, efficient,) and *openness* (e.g. original, inventive, curious).

*Dog Big Five Inventory* (DBFI, [76]; see S5 Appendix; Hungarian translation: [60]; German translation: own translation according to the human questionnaire): The Inventory was adapted to dogs utilizing the human Five Factor Model (FFM) by Gosling et al. [76]. DBFI consists of five scales which are similar or the same as in the human BFI: *energy* (analogue to human *extraversion*), *neuroticism, affection* (analogue to *agreeableness*), *conscientiousness* and *intelligence* (analogue to *openness*). The owners had to score their dogs along 43 items on a 5-point scale ranging from 1 (disagree strongly) to 5 (agree strongly). The internal consistency, inter-observer reliability and external validity of the questionnaire were supported by Gosling et al. [76]. The accuracy of owners’ judgements about their dogs’ personality was supported by the correlation with behaviour ratings of independent observers who observed dogs’ behaviour in a later field-testing session [76].

### Statistical analysis

Scores on the attachment and personality sub-scales were calculated. The internal consistencies of questionnaire scales were acceptable/high: Cronbach’s alphas were between 0.59 and 0.88 (calculated separately for each sub-scale for the German and Hungarian data in SPSS 16.0). The *intelligence, energy* and *affection* sub-scales of the DBFI, and the attachment scales had only moderate internal consistency (Cronbach’s alpha was less than 0.7), but only in one of the datasets (either in the Hungarian or German).

Sum scores of SRD symptoms were significantly higher in dogs that their owners considered as having SRD than in dogs that were considered as not having SRD in both samples (Germany: sum scores in dogs without SRD (median, lower quartile—upper quartile): 9, 9–11, sum scores in dogs with SRD: 15, 12–18; Mann-Whitney test: z = 17.51, p < 0.001; Hungary: sum scores in dogs without SRD: 10, 9–11.25, sum scores in dogs with SRD: 15, 13–17; Mann-Whitney test: z = 11.17, p < 0.001). Because the answer of the owners on the SRD question indicated well the number of SRD symptoms the dog actually showed, and because there is no consensus which symptoms are required for the diagnosis of SRD (see for example [20,45,46,51]), we used the owners’ answer to the SRD question as an indication of SRD in our analyses.

Sample sizes were highly different between the two populations, and all human personality, human attachment and dog personality differed between the two groups (MANOVAs, Table 1). Furthermore some of the Cohens’s d effect sizes for the differences between the two populations were medium (d = 0.5) or almost large (d = 0.8). Therefore we analysed the data separately for Germany and Hungary.

**Table 1:** Owner personality and attachment scale (AAS) and dog personality from German and Hungarian questionnaire data (mean ± sd).

	Germany	Hungary	*d* (95% CI)	Wilks’ λ	*p*
**Owner personality**	*n* = 1185	*n* = 201			
Extraversion	3.56 ±0.672	3.47 ±0.713	0.13 (0.02–0.23)	0.945	< 0.001
Agreeableness	3.56 ±0.504	3.65 ±0.578	0.17 (0.06–0.27)		
Conscientiousness	3.66 ±0.552	3.65 ±0.650	0.02 (-0.08–0.13)		
Neuroticism	2.79 ±0.715	2.74 ±0.767	0.07 (-0.04–0.17)		
Openness	3.53 ±0.579	3.87 ±0.627	0.59 (0.49–0.70)		
**Owner AAS**	*n* = 1185	*n* = 323			
Avoidance	2.29 ±0.800	2.82 ±0.545	0.70 (0.60–0.81)	0.915	< 0.001
Anxiety	2.07 ±0.830	2.15 ±0.771	0.09 (-0.01–0.19)		
**Dog personality**	*n* = 1185	*n* = 201			
Energy	3.86 ±0.601	3.86 ±0.595	0.01 (-0.09–0.12)	0.942	< 0.001
Affection	3.71 ±0.510	4.02 ±0.563	0.60 (0.49–0.71)		
Conscientiousness	3.48 ±0.552	3.56 ±0.553	0.14 (0.04–0.25)		
Neuroticism	2.70 ±0.740	2.38 ±0.802	0.42 (0.32–0.53)		
Intelligence	3.85 ±0.488	3.80 ±0.433	0.10 (0.00–0.21)		

For each scale the Cohen’s *d* effect size and 95% confidence interval (CI) are given. The three measures between the two populations were compared by MANOVAs for which Wilks’ λs and *p*s are given.

Binomial generalized linear models (GLMs) with separation-related disorder (SRD: 0 or 1) as response variable were used. Four sets of models were built. First, the effect of potential confounding variables (age and gender of dog, and age and gender of owner) were investigated in one model set. As the age of dog and the time the dog and the owner were living together were highly correlated in both datasets (Spearman correlations, Germany: r_s_ = 0.882, n = 1075, p<0.001, Hungary: r_s_ = 0.915, n = 205, p<0.001), only the effect of age of dog on SRD was investigated as confounding variable. All four potential confounding variables were included in the initial model and non-significant terms were removed by backward model selection based on likelihood-ratio tests. In Hungary none of the confounding variables had a significant effect on SRD, whereas in Germany the dogs of female and older owners had less often SRD (likelihood-ratio tests, age of owner: *b* = -0.02±0.007, χ^2^ = 6.48, *df* = 1, *p* = 0.011, gender of owner: *b* = -0.79±0.244, χ^2^ = 9.591, *df* = 1, *p* = 0.002).

Second, the effect of human personality, human attachment and dog personality on SRD was investigated in three binomial GLMs. In case of Germany, also the confounding variables having a significant effect in the first model set were included in all models.

Sample sizes were relatively large in both datasets, and this can cause significant results even at small differences. Therefore, we report not only the parameter estimates and their significance from the GLMs, but also the appropriate standardized effect sizes (*r*) and their 95% confidence intervals (CIs) following Nakagawa and Cuthill [77]. Statistical analyses were performed in R using manova and glm functions and purpose built functions for calculation of effect sizes (version: 3.1.0, R Core Team, 2014). Assumptions of statistical models were checked graphically. All p-values are two-tailed except for tests of asymmetric distributions (χ^2^ and F approximation of Wilks’ λ).

## Results

### Ratio of dogs with SRD in the two samples

In the German sample 218 dogs (18.4%, *n* = 1185) were reported to have SRD. In the Hungarian sample this number is 107 dogs (33.1%, n = 323) with SRD which ratio is significantly higher than the ratio of dogs with SRD in the German sample (χ^2^ = 31.7, *df* = 1, *p* < 0.001).

### Effect of human attachment scales (AAS) on SRD in dogs

**Fig 1:**
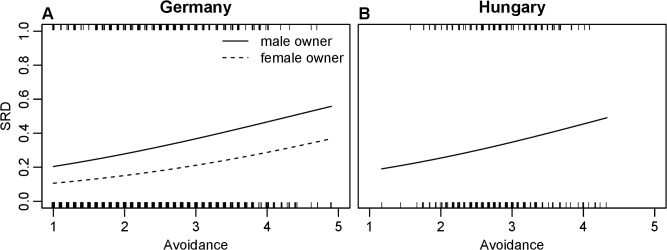
The effect of human avoidance on dog separation disorder (SRD) in German and Hungarian questionnaire data. Lines represent fitted values from binomial GLMs in Table 2. Fitted values were calculated at the mean value of independent variables not shown on the plots. Rugs on the top and bottom of the plots represent the data points with SRD and without SRD, respectively. In case of ties, small amount of random noise were added to visualize the spread of data. A panel represents German, B panel represents Hungarian data.

With increasing *avoidance* score of owners the occurrence of SRD in dogs increased significantly with similar estimated slope (Fig. 1) in the two populations, whereas *anxiety* score of owners had no effect on SRD (Table 2).

**Table 2:** Parameter estimates (± se) from binomial GLMs for the human adult attachment scale (AAS).

	Germany				Hungary			
Parameter	Estimate	*z*	*p*	*r* (95% CI)	estimate	*z*	*p*	*r* (95% CI)
**Avoidance**	0.41 ± 0.106	3.850	< 0.001	0.11 (0.08–0.14)	0.45 ± 0.223	1.999	0.046	0.11 (0.06–0.17)
**Anxiety**	-0.08 ± 0.104	-0.797	0.425	-0.02 (-0.05–0.01)	0.01 ±0.156	0.062	0.951	0.00 (-0.05–0.06)

Wald tests, standardized effect sizes (*r*) and their 95% CI are given. Note that in case of German data gender (*b* = -0.774±0.237, *p* = 0.001) and age of owner (*b* = -0.015±0.007, *p* = 0.031) were also in the model.

### Effect of human personality scales (BFI) on SRD in dogs

None of the human personality scales influenced SRD in dogs significantly in either datasets. (Table 3).

**Table 3:** Parameter estimates (±se) from binomial GLMs for the human personality.

	Germany				Hungary			
Parameter	estimate	*z*	*p*	*r* (95% CI)	estimate	*z*	*p*	*r* (95% CI)
**Extraversion**	-0.01 ± 0.126	-0.109	0.913	0.00 (-0.03–0.03)	-0.17 ± 0.237	-0.737	0.461	-0.05 (-0.12–0.02)
**Agreeableness**	-0.28 ± 0.166	-1.693	0.091	-0.05 (-0.08–-0.02)	0.25 ± 0.305	0.826	0.409	0.06 (-0.01–0.13)
**Conscientiousness**	0.02 ± 0.144	0.161	0.872	0.00 (-0.02–0.03)	-0.43 ± 0.262	-1.655	0.098	-0.12 (-0.19–-0.05)
**Neuroticism**	0.24 ± 0.124	1.923	0.054	0.06 (0.03–0.08)	0.25 ± 0.241	1.023	0.306	0.07 (0.00–0.14)
**Openness**	0.26 ± 0.139	1.831	0.067	0.05 (0.02–0.08)	0.04 ± 0.271	0.165	0.869	0.01 (-0.06–0.08)

Wald tests, standardized effect sizes (*r*) and their 95% CI are given. Note that in case of German data gender (*b* = -0.75±0.240, *p* = 0.002) and age of owner (*b* = -0.01±0.007, *p* = 0.074) were also in the model.

### Effect of dog personality scales (DBFI) on SRD in dogs

**Fig 2:**
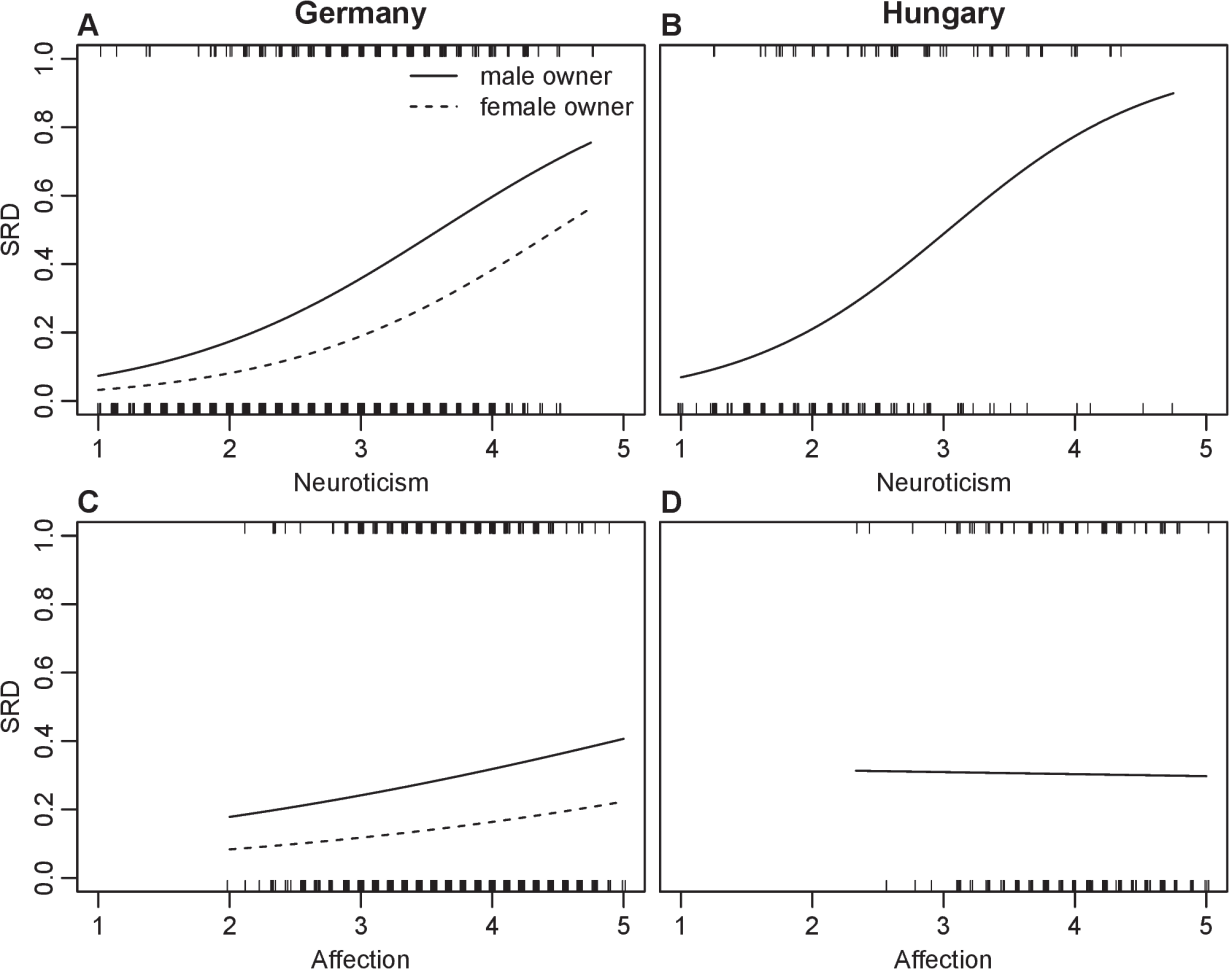
The effect of dog neuroticism and affection on dog separation disorder (SRD) in German and Hungarian questionnaire data. Lines represent fitted values from binomial GLMs in Table 4. For further details see legend of Fig. 1. The panels represent German (A and C) and Hungarian (B and D) data regarding the effect of dogs’ *neuroticism* and *affection*, respectively on dog’s SRD.

In both populations, the more *neurotic* dogs had more often SRD (Table 4). More *affectionate* dogs had more often SRD in Germany, but the effect size was negligible. Furthermore the non-significant slope of this relationship was different in Hungary (Fig. 2). *Energy, conscientiousness* and *intelligence* had no significant effect on dogs’ SRD in either sample.

**Table 4:** Parameter estimates (±se) from binomial GLMs for the dog personality.

	Germany				Hungary			
Parameter	estimate	*z*	*p*	*r* (95% CI)	estimate	*Z*	*p*	*r* (95% CI)
**Energy**	0.07 ±0.156	0.453	0.650	0.01 (-0.02–0.04)	0.42 ±0.328	1.296	0.195	0.09 (0.02–0.16)
**Affection**	0.38 ±0.177	2.167	0.030	0.06 (0.03–0.09)	-0.03 ±0.353	-0.079	0.937	-0.01 (-0.08–0.07)
**Conscientiousness**	-0.31 ±0.165	-1.847	0.065	-0.05 (-0.08–-0.02)	0.19 ±0.352	0.529	0.597	0.04 (-0.03–0.11)
**Neuroticism**	0.98 ±0.129	7.551	< 0.001	0.21 (0.19–0.24)	1.28 ±0.279	4.579	< 0.001	0.31 (0.25–0.37)
**Intelligence**	0.19 ±0.213	0.891	0.373	0.03 (0.00–0.06)	0.31 ±0.432	0.726	0.468	0.05 (-0.02–0.12)

Wald tests, standardized effect sizes (*r*) and their 95% CI are given. Note that in case of German data gender (*b* = -0.869±0.247, *p*<0.001) and age of owner (*b* = -0.007±0.007, *p* = 0.303) were also in the model.

## Discussion

### Effect of human attachment on dogs’ SRD

In accordance with our hypothesis we found that owners scoring higher on self-reported attachment *avoidance* are more likely to have dogs with separation-related disorder. Although we cannot be sure about the direction and causality of this relationship, there are reasons to assume that the owners’ avoidant attachment contributes (at least in part) to the behaviour problem of the dog. This assumption is supported by the analogy between child-parent and dog-owner relationship [4,5] and by the results of the human studies on parent-child attachment and parenting (see Introduction).

We suppose that owners’ attachment style influences their caregiving behaviour toward the dog: they may show a less consistent responsiveness to the dog’s needs. Owners with insecure-avoidant attachment style avoid intimate contacts, closeness and affection [34] and it is possible that they behave in this way not only in their interpersonal relationships but also toward their dogs. Dogs who meet refusal or ignorance of their needs (e.g. need for contact) can learn that they cannot be sure about the availability of the owner. We should note, however, that a recent study did not find a correlation between people’s interpersonal attachment avoidance and avoidance toward their pet, although a matching was found in the case of attachment anxiety [78].

Additionally, avoidant owners may refuse the attachment behaviour of their dog especially in stressful situations. This assumption is also based on the human literature: mothers displaying high levels of avoidance are less responsive when their child is highly distressed, while this pattern is reversed among parents scoring low on avoidance [79]. According to Van Ijzendoorn’s [35], dismissing parents may often refuse the attachment behaviour of their child in stressful situations because such behaviours trigger negative attachment-related memories in them.

As a consequence, avoidant mothers’ children experience higher distress during the stressful event [79]. Similarly, avoidant dog-owners’ refusal of the dog’s attachment behaviour may contribute to the dogs’ stress response to separation escalates. Thus, the owner constitutes neither a secure base (see also [50]) nor a safe haven for them. An insecure attachment develops in the dog that can contribute to SRD.

We found no effect of owners’ attachment *anxiety* on the dog’s SRD, which is in accordance with the findings of the human mother-child attachment studies. Namely, that parental anxious attachment does not lead to deficiency in parental sensitivity/responsiveness, but rather in autonomy support/non-intrusiveness [36,38]. As parental sensitivity seems to play the primary role in developing secure attachment (e.g. [27]) and separation anxiety [25] in the child, it is a logical consequence that *avoidance* in the parent contributes to separation anxiety in the child, but attachment *anxiety* does not. The same may be true for owners and dogs.

### Effect of human personality on dogs’ SRD

The results do not support our hypothesis that owners with higher neuroticism are more likely to have dogs with SRD. It is surprising because in the human literature association was found between mothers’ (or parents’) neuroticism and anxiety disorder and children’s less secure attachment, behaviour problems and separation anxiety disorder [42,80,81]. In case of dogs, owners’ neuroticism correlated with dogs’ neuroticism [60] (which was found also to predict dogs’ SRD in the present study, see later) and with the degree of the dog’s displacement activities [56]. In the light of these results one can assume that owners’ neuroticism can also contribute to separation-related disorder in the dog. Although the effect was almost significant (p = 0.054) in the German sample, we cannot verify our hypothesis. Maybe other factors should be investigated in future studies that can account for the weakness of the relationship.

### Effect of dogs’ personality on SRD

In accordance with our hypothesis, we found in both samples that more *neurotic* dogs had more often SRD. *Neurotic* dogs are prone to stress (and to other negative emotions) in any situation including separation situations. This is in accordance with Mendl et al’s [53] study which suggests that SRD dogs are generally in a negative affective state. This proneness to negative emotions and distress can be the result (at least partly) of the attachment problem as it is supported by some evidence in the case of humans (e.g. [82]). According to Bowlby [15] the attachment relationship directly influences the infant’s capacity to cope with stress by impacting the maturation of the „control system” of the infant’s mind. Fischer-Mamblona [83] argues also that the lack of a primary attachment object may cause an immense escape motivation both in humans and in animals.

In a previous study [50] we found that SRD dogs did not show more affection toward the owner and the same was found by Parthasarathy and Crowell-Davis [55]. We interpreted these results as an argument against the commonly hold belief that SRD dogs are “hyper-attached” to the owner [50]. In the present study our results are mixed: although in the Hungarian sample we did not find any effect of *affection* on dogs’ SRD, in the German sample more *affectionate* dogs had more often SRD (although the effect size was negligible). Interestingly, the non-significant slope of this relationship was different in Hungary. The reason of this difference is unclear and needs to be investigated in future research.

### Ratio of dogs with SRD in the two samples

Hungarian owners reported SRD in their dog in a significantly higher ratio than German owners. According to a recent survey which screened 1201 dog owners with 1960 dogs across the United States 13% of the dogs had separation anxiety [84]. Thus, the ratio seen in the German sample is similar to the American one. In a previous, experimental study [50] in Hungary we experienced the same high ratio of SRD in a rather limited sample (15 out of 44 dogs, 34%) as in the present Hungarian sample. The reason of this difference in the prevalence of SRD between nations is still unclear and needs to be investigated in future research.

### Limitations of the study

The procedure of this study does not exclude some alternative explanations of the results. We measured owners’ opinion about their dogs’ SRD, thus, the “diagnosis” of SRD might mirror only the subjective view of the owners. In this case an alternative explanation of the results is that insecure owners might see their dogs differently than secure ones: it is possible that they consider them to be more problematic than securely attached owners. This would be in accordance with the finding that neurotic owners regard their dog’s phobic behaviour more as a problem although the degree of the phobic behaviour did not correlate with owner’s neuroticism [56]. However, our previous finding suggests the opposite: owners perceive correctly the separation and greeting behaviour of their dog and dogs which were reported by the owner to have SRD showed indeed more distress during separation in a behaviour test [50].

It is also possible that owners’ insecure attachment is not a triggering factor of the dogs’ SRD but it influences the selection of a puppy/ adult dog and/or a dog breed. This would be in accordance also with the psychological „similarity-attraction hypothesis” which suggests that the more similar two individuals are, the higher the attraction between them [85,86]. Turcsán et al. [60] found positive correlations in all the five personality traits using the Big Five Inventory between owners and dogs living in single-dog households (similarly to those found in close human social relationships, e.g. [87]). This would suggest that owners select dogs that are similar to themselves, either at the individual or at the breed level. People with higher avoidance may have a different personality than less avoidant people [43], so avoidant owners may chose puppies/a breed with different personality than secure owners; and the personality of the dog can contribute to the later attachment security and SRD. Based on the results of the present study we cannot exclude this possibility. Comparison of personality of puppies chosen by owners with different attachment style could clarify this issue.

Another limitation of the study might be that we did not include in the analysis many potential contributors to SRD. Factors such as breed [88], genetic predisposition to stress [89], other dogs in the household [20], or training experiences of the dog [61] can all have an effect on the occurrence of SRD. However, our aim was not to assess all these contributors. There are some studies (e.g. [45]) aimed to investigate a wide range of potential risk factors to SRD, but our purpose was to investigate the effect of a so far unobserved contributor to the disorder.

The experimental set-up used in our previous study (Konok et al., 2011), namely the Separation and Greeting Test could be used in future research to validate the results. There we showed that dogs with owner-reported SRD behaved differently than dogs without owner-reported SRD, and we proposed that this behaviour test along with the questionnaire could be used for screening SRD. An index could be calculated from those behavioural variables which SRD dogs differed significantly from non-SRD dogs. Videos of the dogs might be evaluated by dog-experts to further validate the behavioural and the questionnaire „diagnosis”.

## Conclusions

Our results suggest that owners’ attachment avoidance may facilitate the development of SRD in dogs in addition to several possible factors (see above). We assume that avoidant owners’ are less responsive to the dog’s needs and do not provide a secure base for the dog when needed and as a result dog form an insecure attachment and develop SRD.

We believe that our findings are important for both theoretical and practical reasons. The results throw new lights upon the possible role of the owner in the emergence of SRD in family dogs. Owners of dogs that may be prone to develop SRD could be made aware about the need of consistent and reliable responsiveness toward the dog. In addition, in the study of parental effects on attachment patterns, the owner-dog attachment model has the advantage that there is no underlying genetic factor which can be confounding in human studies.

Finally, these observations may present the basis for new therapeutic approaches of SRD in dogs. So far the management of the problem consists of environmental control, behaviour modification/training, and medication [20]. Based on the results new approaches to behaviour therapy in dogs can be developed that include the improvement of the self-knowledge of the owner, or even the modification of his/her inner working models of attachment [90,91], hereby integrating psychotherapy and dog behaviour therapy.

## Supporting Information

S1 Appendix Demographic questions. (DOC)

S2 Appendix Separation Behaviour Questionnaire. (DOC)

S3 Appendix Adult Attachment Scale. (DOC)

S4 Appendix Big Five Inventory. (DOC)

S5 Appendix Dog Big Five Inventory. (DOC)
