# Supplementary material for: Influence of Owners’ Attachment Style and Personality on Their Dogs’ (Canis familiaris) Separation-Related Disorder
Source: PLoS One. 2015 Feb 23;10(2):e0118375. doi: 10.1371/journal.pone.0118375 (PMC4338184; doi:10.1371/journal.pone.0118375)
Supplement: S1 Appendix — (DOC) [file pone.0118375.s001.doc]

Appendix S1

**Demographic questions**

Dog's name:

Dog's breed:

Dog’s age (in years):

Dog's sex:

How long have you been living with your dog? (in years)

Your sex:

Your age (in years):
