# Supplementary material for: Influence of Owners’ Attachment Style and Personality on Their Dogs’ (Canis familiaris) Separation-Related Disorder
Source: PLoS One. 2015 Feb 23;10(2):e0118375. doi: 10.1371/journal.pone.0118375 (PMC4338184; doi:10.1371/journal.pone.0118375)
Supplement: S2 Appendix — (DOC) [file pone.0118375.s002.doc]

Appendix S2

Separation Behaviour Questionnaire

Does your dog have separation anxiety, that is, any behaviour problem related to leaving it alone? Yes/ No

Do you experience the following behaviours in your dog in those situations when you leave it alone or during the previous minutes (when the dog can already see that you will leave it alone)? Please, mark how frequently these behaviours happen.

1------------------2-----------------3-----------------4-----------------5

Never Seldom Often Always/ I don’t know

almost always

Whining

Barking

Howling

Intensive salivation

Urination in the flat/ at inappropriate places

Defecation in the flat/ at inappropriate places

Destruction of objects/ parts of the flat (scratching, chewing, etc.)

Trembling, shaking

Agitation, restlessness, pacing
