# Supplementary material for: Influence of Owners’ Attachment Style and Personality on Their Dogs’ (Canis familiaris) Separation-Related Disorder
Source: PLoS One. 2015 Feb 23;10(2):e0118375. doi: 10.1371/journal.pone.0118375 (PMC4338184; doi:10.1371/journal.pone.0118375)
Supplement: S3 Appendix — (DOC) [file pone.0118375.s003.doc]

Appendix S3

Adult Attachment Scale

Please read each of the following statements and rate the extent to which it describes your feelings about close relationships.

Please use the scale below by placing a number between 1 and 5 in the space provided to the right of each statement.

1---------------2---------------3---------------4---------------5

Not at all characteristic

of me

Very characteristic

of me

(1) I find it relatively easy to get close to others. ________

(2) I do not worry about being abandoned. ________

(3) I find it difficult to allow myself to depend on others. ________

(4) In relationships, I often worry that my partner does not really love me. ________

(5) I find that others are reluctant to get as close as I would like. ________

(6) I am comfortable depending on others. ________

(7) I do not worry about someone getting too close to me. ________

(8) I find that people are never there when you need them. ________

(9) I am somewhat uncomfortable being close to others. ________

(10) In relationships, I often worry that my partner will not want to stay with me. ________

(11) I want to merge completely with another person. ________

(12) My desire to merge sometimes scares people away. ________

(13) I am comfortable having others depend on me. ________

(14) I know that people will be there when I need them. ________

(15) I am nervous when anyone gets too close. ________

(16) I find it difficult to trust others completely. ________

(17) Often, partners want me to be closer than I feel comfortable being. ________

(18) I am not sure that I can always depend on others to be there when I need them. ________
