# Supplementary material for: Influence of Owners’ Attachment Style and Personality on Their Dogs’ (Canis familiaris) Separation-Related Disorder
Source: PLoS One. 2015 Feb 23;10(2):e0118375. doi: 10.1371/journal.pone.0118375 (PMC4338184; doi:10.1371/journal.pone.0118375)
Supplement: S4 Appendix — (DOC) [file pone.0118375.s004.doc]

Appendix S4

Big Five Inventory

Here are a number of characteristics that may or may not apply to you. Please write a number next to each statement to indicate the extent to which you agree or disagree with that statement.

| 1 | 2 | 3 | 4 | 5 |
| --- | --- | --- | --- | --- |
| disagree  strongly | disagree  a little | neither agree  nor disagree | agree  a little | agree  strongly |

I see myself as someone who...

| 1 | ........ is talkative | 23 | ........ tends to be lazy |
| --- | --- | --- | --- |
| 2 | ........ tends to find fault with others | 24 | ........ is emotionally stable, not easily upset |
| 3 | ........ does a thorough job | 25 | ........ is inventive |
| 4 | ........ is depressed, blue | 26 | ........ has an assertive personality |
| 5 | ........ is original, comes up with new ideas | 27 | ........ can be cold and aloof |
| 6 | ........ is reserved | 28 | ........ perseveres until the task is finished |
| 7 | ........ is helpful and unselfish with others | 29 | ........ can be moody |
| 8 | ........ can be somewhat careless | 30 | ........ values artistic, aesthetic experiences |
| 9 | ........ is relaxed, handles stress well | 31 | ........ is sometimes shy, inhibited |
| 10 | ........ is curious about many different things | 32 | ........ is considerate and kind to almost everyone |
| 11 | ........ is full of energy | 33 | ........ does things efficiently |
| 12 | ........ starts quarrels with others | 34 | ........ remains calm in tense situations |
| 13 | ........ is a reliable worker | 35 | ........ prefers work that is routine |
| 14 | ........ can be tense | 36 | ........ is outgoing, sociable |
| 15 | ........ is ingenious, a deep thinker | 37 | ........ is sometimes rude to others |
| 16 | ........ generates a lot of enthusiasm | 38 | ........ makes plans and follows through with them |
| 17 | ........ has a forgiving nature | 39 | ........ gets nervous easily |
| 18 | ........ tends to be disorganized | 40 | ........ likes to reflect, play with ideas |
| 19 | ........ worries a lot | 41 | ........ has few artistic interests |
| 20 | ........ has an active imagination | 42 | ........ likes to cooperate with others |
| 21 | ........ tends to be quiet | 43 | ........ is easily distracted |
| 22 | ........ is generally trusting | 44 | ........ is sophisticated in art, music, or literature |
