# Supplementary material for: Influence of Owners’ Attachment Style and Personality on Their Dogs’ (Canis familiaris) Separation-Related Disorder
Source: PLoS One. 2015 Feb 23;10(2):e0118375. doi: 10.1371/journal.pone.0118375 (PMC4338184; doi:10.1371/journal.pone.0118375)
Supplement: S5 Appendix — (DOC) [file pone.0118375.s005.doc]

Appendix S5

Dog Big Five Inventory

Here are a number of characteristics that may or may not apply to your dog. Please write a number next to each statement to indicate the extent to which you agree or disagree with that statement.

| 1 | 2 | 3 | 4 | 5 |
| --- | --- | --- | --- | --- |
| disagree  strongly | disagree  a little | neither agree  nor disagree | agree  a little | agree  strongly |

I see my dog as an individual who...

| 1 | ........ is talkative, vocal | 23 | ........ tends to be lazy |
| --- | --- | --- | --- |
| 2 | ........ is disagreeable, difficult to please | 24 | ........ is emotionally stable, not easily upset |
| 3 | ........ does things thoroughly | 25 | ........ is inventive, finds new ways to get his/her way |
| 4 | ........ is down, depressed, blue | 26 | ........ has an assertive personality |
| 5 | is original, comes up with new ways of  ........ doing things | 27 | ........ can be cold and aloof |
| 6 | ........ is reserved | 28 | ........ perseveres until the task is finished |
| 7 | ........ is helpful and unselfish | 29 | ........ can be moody |
| 8 | ........ can be somewhat careless | 30 | ........ appreciates sensory experiences |
| 9 | ........ is relaxed, handles stress well | 31 | ........ is sometimes shy, inhibited |
| 10 | ........ is curious about many different things | 32 | ........ is considerate and kind |
| 11 | ........ is full of energy | 33 | ........ does things efficiently |
| 12 | ........ starts quarrels with others | 34 | ........ remains calm in tense situations |
| 13 | ........ is a reliable dog | 35 | ........ enjoys learning and doing new things |
| 14 | ........ can be tense | 36 | ........ is outgoing, sociable |
| 15 | ........ appears contemplative, thoughtful | 37 | ........ is sensitive to the needs and feelings of others |
| 16 | ........ shows a lot of enthusiasm | 38 | ........ is planful, determined |
| 17 | ........ has a forgiving nature | 39 | ........ gets nervous easily |
| 18 | ........ tends to be disorganized | 40 | ........ appears to “reflect,” mull things over |
| 19 | ........ worries a lot | 41 | ........ is cooperative |
| 20 | ........ is unimaginative, dull | 42 | ........ is easily distracted |
| 21 | ........ tends to be quiet | 43 | ........ is sophisticated |
| 22 | ........ is generally trusting |  |  |
